# Association between *GRIN3A* Gene Polymorphism in Kawasaki Disease and Coronary Artery Aneurysms in Taiwanese Children

**DOI:** 10.1371/journal.pone.0081384

**Published:** 2013-11-22

**Authors:** Ying-Ju Lin, Jeng-Sheng Chang, Xiang Liu, Chien-Hui Hung, Ting-Hsu Lin, Shao-Mei Huang, Kuan-Teh Jeang, Chia-Yen Chen, Chiu-Chu Liao, Cheng-Wen Lin, Chih-Ho Lai, Ni Tien, Yu-Ching Lan, Mao-Wang Ho, Wen-Kuei Chien, Jin-Hua Chen, Yu-Chuen Huang, Hsinyi Tsang, Jer-Yuarn Wu, Chien-Hsiun Chen, Li-Ching Chang, Fuu-Jen Tsai

**Affiliations:** 1 Department of Medical Research, China Medical University Hospital, Taichung, Taiwan; 2 School of Chinese Medicine, China Medical University, Taichung, Taiwan; 3 Department of Pediatrics, China Medical University Hospital, Taichung, Taiwan; 4 Molecular Virology Section, Laboratory of Molecular Microbiology, National Institute of Allergy and Infectious Diseases, National Institutes of Health, Bethesda, Maryland, United States of America; 5 Graduate Institute of Clinical Medical Science, Chang-Gung University, Chiayi, Taiwan; 6 Viral Biochemistry Section, Laboratory of Molecular Microbiology, National Institute of Allergy and Infectious Diseases, National Institutes of Health, Bethesda, Maryland, United States of America; 7 Department of Medical Laboratory Science and Biotechnology, China Medical University, Taichung, Taiwan; 8 Department of Microbiology, School of Medicine, China Medical University, Taichung, Taiwan; 9 Department of Health Risk Management, China Medical University, Taichung, Taiwan; 10 Section of Infectious Diseases, Department of Internal Medicine, China Medical University Hospital, Taichung, Taiwan; 11 Biostatistics Center, China Medical University, Taichung, Taiwan; 12 Biostatistics Center, Taipei Medical University, Taichung, Taiwan; 13 The Laboratory of Molecular Immunogenetics, National Institute of Allergy and Infectious Diseases, National Institutes of Health, Bethesda, Maryland, United States of America; 14 Institute of Biomedical Sciences, Academia Sinica, Taipei, Taiwan; 15 Asia University, Taichung, Taiwan; National Taiwan University Hospital, Taiwan

## Abstract

Kawasaki disease (KD) is pediatric systemic vasculitis with the classic complication of coronary artery aneurysm (CAA). It is the leading cause of acquired cardiovascular diseases in children. Some severe cases present with multi-organ involvement or neurological dysfunction. To identify the role of the glutamate receptor, ionotropic, *N*-methyl-d-aspartate 3A (*GRIN3A*) in KD, we investigated genetic variations in *GRIN3A* in a Taiwanese cohort of 262 KD patients (76 with and 186 without CAA complications). We used univariate and multivariate regression analyses to identify the associations between clinical characteristics and *GRIN3A* genetic variations in KD. According to univariate regression analysis, CAA formation in KD was significantly associated with fever duration (*p* < 0.0001), first Intravenous immunoglobulin (IVIG) used (days after day one of fever) (*p* < 0.0001), and the *GRIN3A* (rs7849782) genetic variant (*p* < 0.001). KD patients with GG+GC genotype showed a lower rate of developing CAA (GG+GC genotype: odds ratio = 0.26; 95% CI = 0.14–0.46). Significant associations were identified between KD with CAA complication and the *GRIN3A* (rs7849782) genetic variant by using multivariate regression analysis. Specifically, significant correlations were observed between KD with CAA complications and the presence of GG+GC genotypes for the *GRIN3A* rs7849782 single-nucleotide polymorphism (full model: odds ratio = 0.25; 95% CI = 0.14–0.46). Our results suggest that a polymorphism of the *GRIN3A* gene may play a role in KD pathogenesis.

## Introduction

Kawasaki disease (KD) is acute systemic vasculitis with the classic complication of coronary artery aneurysm (CAA). It is one of the leading causes of acquired cardiovascular diseases in children [1–6]. Vascular inflammation disrupts the balance between endothelial destruction and regeneration. Endothelial dysregulation leads to increased wall vulnerability accompanied by blood leaks and artery dilation [7,8]. These lesions can occur in different organs [9]. Some severe cases present with additional complications involving multiple organs or neurological dysfunction [10–12].

Several genome-wide association screenings have indicated that host genetic variants play important roles in the disease susceptibility of KD [13–18]. In the European population, loci of *ZFHX3*, *NAALADL2*, *PPP1R14C*, *TCP1*, *LNX1*, *CAMK2D*, *CSMD1*, *FCGR2A*, *MIA/RAB4B*, and *ITPKC* harboring genetic variants have been reported as susceptibility loci for KD [13,17]. These genes are related to immune activation, inflammation, apoptosis and cardiovascular pathology. In the Taiwanese population, genetic variants in *COPB2*, *ERAP1*, *IGHV*, *BLK* and *CD40* are associated with KD susceptibility [15,16]. These genes have been implicated in immune activation, inflammation, T cell receptor signaling, regulation of proinflammatory cytokines, and antibody-mediated immune responses. Interestingly, in the Japanese population, loci of *FAM167A-BLK*, *CD40*, *FCGR2A*, and *ITPKC* harboring genetic variants are also reported as the risk loci for KD susceptibility [18,19]. Genetic studies on CAA formation in KD, performed using candidate gene approach, have shown the involvement of genetic variants in *MICB*, *PELI1*, *CASP3*, *CD40*, *MMP-3*, *MMP-12*, *HLA-B associated* transcript 2, 3, and 5, *ITPR3*, *HLA-E*, *HLA-G*, *ITPKC*, *IL-10*, and *angiotensin I converting enzyme* (*ACE*) genes et al [4,14,19–28]. These studies identified candidate genes involved in the immune-regulatory responses and cardiovascular-related pathogenesis that contribute to susceptibility to and/or formation of CAA in KD. 

Expression of the N-methyl-d-aspartate (NMDA) receptor has been described in the barrier forming endothelial cells and the neuroepithelium [29–31]. Additionally, activation of the NMDA receptor can decrease the effectiveness of the endothelial barrier by increasing cytosolic Ca^2+^ formation and junction disorganization [30–32]. Activation of the NMDA receptor can further activate endothelial cell inflammation [33]. Anti-NMDA receptor autoantibodies from SLE patients were reported to activate endothelial cells to express adhesion molecules and secrete inflammatory cytokines and chemokines. During the acute stage of KD, activated vascular endothelium cells with increased serum proinflammatory cytokines are involved in vessel inflammation and injury [34,35]. Injured vascular tissues show subendothelial edema, vascular damage, gap formation, and fenestration of endothelial cells and contribute to the pathogenesis of this disorder. The roles of the NMDA receptor in neurovascular interactions, barrier regulation, and vascular inflammation in systemic vasculitis such as KD are not well understood.

In this study, we examined the association between glutamate receptor, ionotropic, *N*-methyl-d-aspartate 3A (*GRIN3A*) genetic variants and KD in a Taiwanese cohort of 262 KD patients (76 with CAA and 186 with no CAA complications). The relationship between clinical characteristics and aneurysm formation in patients with *GRIN3A* genetic variations was evaluated.

## Materials and Methods

### Ethical statement

 This study was approved by the Human Studies Committee of China Medical University Hospital. Written informed consent was obtained from either the parents or the participants. All parents/guardians of minors provided written informed consent.

### Study subjects

 Unrelated individuals fulfilling the diagnostic criteria of KD (n = 262) were identified and enrolled in the study from the Department of Pediatrics at China Medical University Hospital in Taichung, Taiwan [36–40]. A total of 262 individuals (174 males and 88 females) with an average age at diagnosis of 1.75 ± 1.61 years were included in the study (Table 1). All patients were diagnosed according to KD criteria [36,38], including fever lasting 5 or more days and at least 4 of the following symptoms: (1) changes in extremities (e.g., erythema, edema, or desquamation), (2) bilateral conjunctivitis, (3) polymorphous rash, (4) cervical lymphadenopathy, and (5) changes in lips or oral cavity (e.g., pharyngeal erythema, dry/fissured or swollen lips, strawberry tongue). All the KD patients were treated with IVIG in the acute stage before their development of coronary artery aneurysms. All patients had regular echocardiography examinations during the acute stage, 2 months after onset, 6 months after onset, and once per year thereafter. According to the Japanese Ministry of Health criteria, CAA was identified when either the right or the left coronary artery showed an increase in the dilated diameter by > 3 mm in children below 5 years of age or by > 4 mm in older children [36]. We categorized CAAs from grade B to grade F according to CAA severity grade: CAA- indicates patients with no complications; CAA+ B grade indicates patients with CAA, but who showed remission in 2 months; CAA+ C grade indicates patients with CAA persistence until 2 months, but with remission in 6 months; CAA+ D grade indicates patients with CAA persistence until 6 months; CAA+ E grade indicates patients with giant CAA( ≥ 8mm) or severe stenosis or occlusion; CAA+ F grade indicates patients with sudden death (Table 1). Only Han Chinese individuals, who account for 98% of Taiwanese residents, were recruited. The ethnic background was assigned based on the results of self-report questionnaires.

**Table 1:** Clinical characterististics of CAA-positive and CAA-negative individuals with Taiwanese Kawasaki disease.

	**Kawasaki disease**			***p* value**
	**CAA-**		**CAA+**	
Number	186		76	
Age at Kawasaki disease diagnosis (years)	1.70±1.51		1.86±1.78	0.389
Gender				
Male (Number (%))	120 (64.52%)		54 (71.05%)	0.387**^*a*^**
Female (Number (%))	66 (35.48%)		22 (28.95%)	
Fever duration (days)	7.48±2.67		10.61±4.90	< 0.0001**^*b*^**
1st IVIG used time (days after day one of fever)	6.31±2.24		8.14±4.10	< 0.0001**^*b*^**
CAA severity grade				
B (Number (%))	–		52 (68.42%)	
C (Number (%))	–		9 (11.84%)	
D (Number (%))	–		12 (15.79%)	
E (Number (%))	–		3 (3.95%)	
F (Number (%))	–		0 (0%)	

CAA, Coronary artery aneurysm; IVIG, Intravenous immunoglobulin.

CAA was identified when either the right or the left coronary artery showed an increase in the dilated diameter by > 3 mm in children below 5 years of age or by > 4 mm in older children [35].

CAA severity grade: CAA- indicates patients with no complications; CAA+ B grade indicates patients with CAA, but who showed remission in 2 months; CAA+ C grade indicates patients with CAA persistence until 2 months, but with remission in 6 months; CAA+ D grade indicates patients with CAA persistence until 6 months; CAA+ E grade indicates patients with giant CAA ( ≥ 8 mm) or severe stenosis or occlusion; CAA+ F grade indicates patients with sudden death.

*a* Chi-square test.

*b* student-t test.

### SNP genotyping

**Figure 1:**
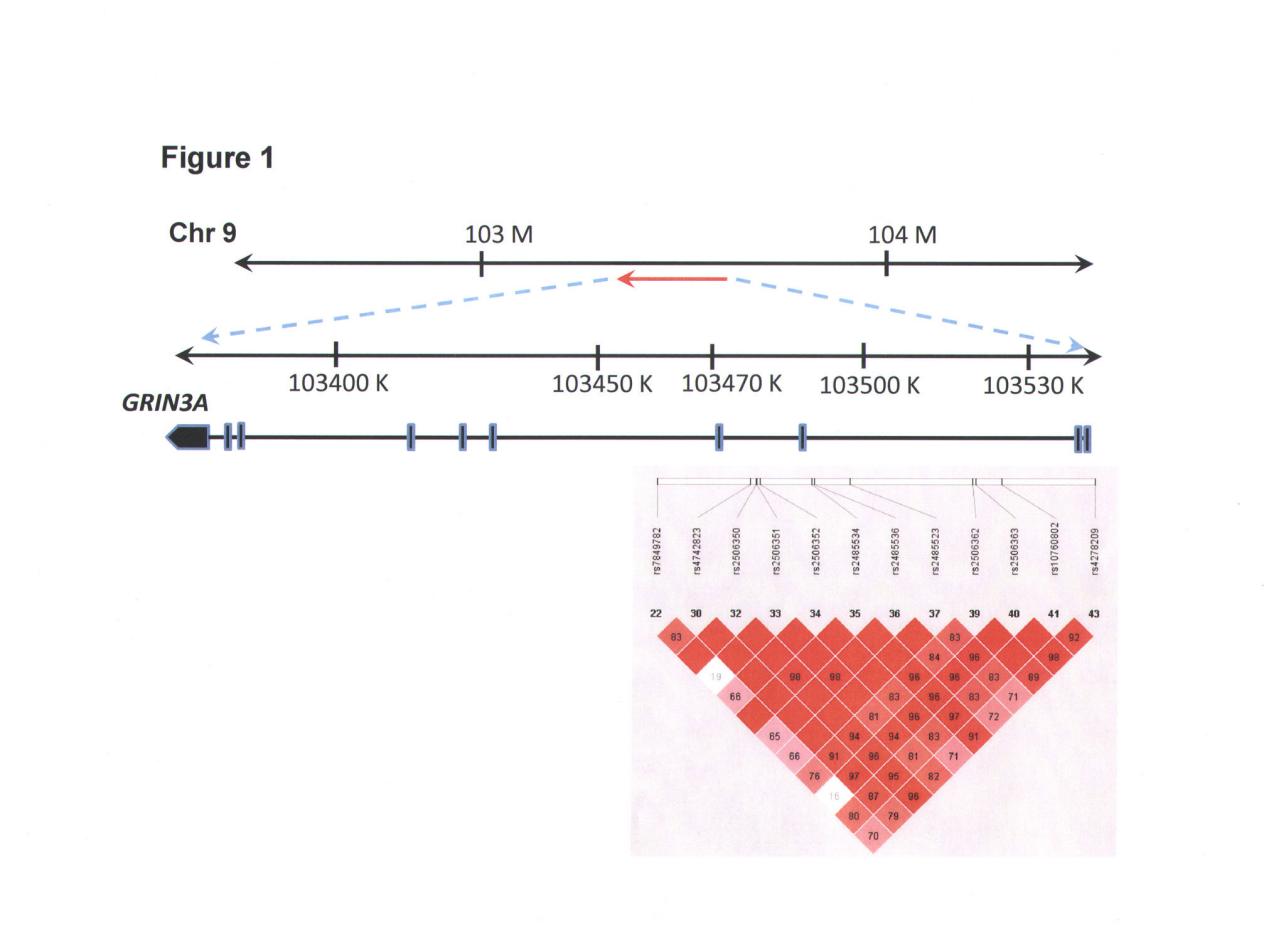
Single-nucleotide polymorphisms (SNPs) analyzed and linkage disequilibrium (LD) pattern of the GRIN3A gene used in this study. Genomic location of the SNPs present on chromosome 9q31.1. Linkage disequilibrium (LD) blocks in the GRIN3A gene estimated using HAPLOVIEW software [43]. Pairwise D′ values (%) are indicated in squares; red indicates linkage disequilibrium (D′ = 1, logarithm of odds (LOD) ≥ 2).

Twelve single-nucleotide polymorphisms (SNPs) from *GRIN3A* were selected from the NCBI SNP database and HAPMAP website (Figure 1 and Table 2) [41–43]. Selection criteria for including SNPs in the analysis were a minimum allele frequency of >0.05 in the Han Chinese population and Hardy-Weinberg equilibrium (HWE; *p* > 0.05). A summary of information regarding SNPs in the *GRIN3A* gene (location, position, rs number, and genotype) is listed in Table 2. Briefly, genomic DNA was extracted from peripheral blood leukocytes according to standard protocols (Genomic DNA kit; Qiagen, Hilden, Germany). SNPs were genotyped using a custom-designed VeraCode GoldenGate Genotyping Assay System (Illumina) [44]; genotyping was performed as described at http://www.illumina.com/.

**Table 2:** Effect of *GRIN3A* gene SNPs on the CAA formation in Taiwanese Kawasaki disease patients.

**SNP**	**SNP Chromosome**	**Cytoband**	**Physical Position**	**Nearest Genes**		**CAA-**	**CAA+**		
						**No. (%)**	**No. (%)**	***p* value**	**Odds ratio (95% CI)**
rs7849782	9	q31.1	103467085	*GRIN3A*	GG+GC	148 (79.6)	38 (50.0)	< 0.001*	0.26 (0.14-0.46)
					CC	38 (20.4)	38 (50.0)		1
rs4742823	9	q31.1	103481593	*GRIN3A*	CC+CT	115 (61.8)	39 (51.3)	0.118	0.65 (0.38-1.11)
					TT	71 (38.2)	37 (48.7)		1
rs2506350	9	q31.1	103482467	*GRIN3A*	TT+TA	67 (36.0)	20 (26.3)	0.132	0.63 (0.35-1.15)
					AA	119 (64.0)	56 (73.7)		1
rs2506351	9	q31.1	103482557	*GRIN3A*	CC+CT	120 (64.5)	48 (63.2)	0.835	0.94 (0.54-1.64)
					TT	66 (35.5)	28 (36.8)		1
rs2506352	9	q31.1	103483140	*GRIN3A*	AA+AG	119 (64.0)	45 (59.2)	0.470	0.82 (0.47-1.41)
					GG	67 (36.0)	31 (40.8)		1
rs2485534	9	q31.1	103491159	*GRIN3A*	TT+TA	110 (60.4)	32 (42.7)	0.010	0.49 (0.28-0.84)
					AA	72 (39.6)	43 (57.3)		1
rs2485536	9	q31.1	103491461	*GRIN3A*	AA+AG	119 (64.0)	46 (61.3)	0.689	0.89 (0.51-1.55)
					GG	67 (36.0)	29 (38.7)		1
rs2485523	9	q31.1	103497057	*GRIN3A*	GG+GA	119 (64.0)	45 (59.2)	0.470	0.82 (0.47-1.41)
					AA	67 (36.0)	31 (40.8)		1
rs2506362	9	q31.1	103516083	*GRIN3A*	AA+AG	120 (64.9)	39 (51.3)	0.043	0.57 (0.33-0.98)
					GG	65 (35.1)	37 (48.7)		1
rs2506363	9	q31.1	103516551	*GRIN3A*	CC+CG	118 (63.4)	48 (63.2)	0.966	0.99 (0.57-1.72)
					GG	68 (36.6)	28 (36.8)		1
rs10760802	9	q31.1	103520656	*GRIN3A*	TT+TC	118 (63.4)	38 (50.0)	0.045	0.58 (0.34-0.99)
					CC	68 (36.6)	38 (50.0)		1
rs4278209	9	q31.1	103535011	*GRIN3A*	AA+AG	118 (63.8)	40 (52.6)	0.095	0.63 (0.37-1.08)
					GG	67 (36.2)	36 (47.4)		1

*GRIN3A*, glutamate receptor, ionotropic, N-methyl-D-aspartate 3A; SNP, single nucleotide polymorphism; CAA, Coronary artery aneurysm; CI, confidence interval.

p-values were obtained by chi-square test.

Bold, emphasizing statistical significance was considered as *p* value <0.0042 (0.05/12).

Primers and probes were designed using Custom VeraCode GoldenGate Genotyping Assay System software. Multiplex PCR cycles were performed with 144-plex VeraCode SNP arrays for 480 samples and genotype analyses were performed using custom 96-plex SAM arrays for 96 samples. Genotype calls were automatically generated using GenCall software version 3.1.3. We individually assessed eight VeraCode runs for intra-plate inconsistencies, such as variations in fluorescence intensities. Genotype cluster plots generated by individual VeraCode and SAM assays were visually inspected for call quality. Plots that appeared to be “unusually” clustered (i.e., those that did not match the predicted spread in terms of software-generated HWE or distance between clusters [θ]) were further investigated by selecting samples via direct Sanger sequencing for genotype confirmation. Samples were sequenced using Big Dye Terminator v3.1 (AB, Foster City, CA, USA) according to the manufacturer’s guidelines and sequenced using an AB 3730 genetic analyzer.

### Analysis of haplotype blocks

Based on HAPLOVIEW software, we used Lewontin D′ measure to estimate the intermarker coefficient of linkage disequilibrium (LD) of patients [43]. The confidence interval of LD was estimated using a resampling procedure and was used to construct haplotype blocks [45].

### Statistical analysis

Unless otherwise indicated, data were expressed as the mean ± standard deviation (SD) for continuous variables. The unpaired Student’s *t* test was used to compare groups (Table 1). Genotypes were obtained by direct counting followed by allele frequency calculations (Table 2). χ^2^ test was used to identify differences in categorical variables, and odds ratios (OR) and 95% confidence intervals (CI) were calculated for the factors under consideration. Forward stepwise multivariate regression analyses were performed to identify factors contributing independently to CAA formation in KD. All statistical analyses were performed using SPSS (v12.0) for Windows.

## Results

### Association between clinical characteristics and KD with CAA formation

The characteristics and clinical profiles of KD patients included in the study are summarized in Table 1. To identify clinical risk factors for CAA formation in KD, patients were divided into 2 groups: 76 with and 186 without CAA complications. Statistically significant differences were observed for fever duration (*p* < 0.0001) and first IVIG used (days after day one of fever) (*p* < 0.0001).

### Association between polymorphisms of the GRIN3A gene and KD with CAA formation in the Han Chinese population in Taiwan

To identify genetic variants linked to KD with CAA formation, we genotyped the *GRIN3A* gene in this study (Tables 1 and 2).

The genetic location of *GRIN3A* is shown in Figure 1; all SNPs were in HWE and showed a successful genotyping frequency of >99%. The LD structure of this region was also established and one haplotype block was determined. This block contained 12 SNPs and included 4 *GRIN3A* exons.

Genotype and genotype frequency data for 12 SNPs are shown in Table 2. A statistically significant difference was observed for the *GRIN3A* (rs7849782) genetic variant (*p* < 0.001). The frequency of individuals carrying the GG+GC genotypes of *GRIN3A* (rs7849782) was 50.0% for KD with CAA and 79.6% for KD without CAA complications. KD patients with GG+GC genotypes showed a lower rate of CAA development (GG+GC genotype: odds ratio = 0.26; 95% CI = 0.14–0.46).

### GRIN3A genetic factor for CAA formation in KD by using multivariate regression analysis

According to the results of univariate regression analysis, statistically significant factors associated with CAA formation in KD were noted for fever duration (*p* < 0.0001), first IVIG used (days after day one of fever) (*p* < 0.0001), and *GRIN3A* (rs7849782) genetic variant (*p* < 0.001) (Tables 1 and 2). To determine the genetic role of *GRIN3A*, we used multivariate regression analyses to identify the associations between clinical characteristics and *GRIN3A* genetic variations in KD. As shown in Table 3, after adjusting for these potential factors, significant associations between KD with CAA complications and the *GRIN3A* (rs7849782) genetic variant were observed. Specifically, significant correlations were observed between KD with CAA complications and the presence of GG+GC genotypes for the *GRIN3A* rs7849782 SNP (full model: odds ratio = 0.25; 95% CI = 0.14–0.46).

**Table 3:** Association of *GRIN3A* genetic variants with CAA formation risk in Taiwanese Kawasaki disease by multivariate regression analysis.

***GRIN3A* genetic variants**	**Odds ratio**	**95% CI**	***p* value**
**Adjusted by Fever duration (days)**			
rs7849782	0.25	0.25 (0.14-0.46)	< 0.001*
rs4742823	0.66	0.66 (0.38-1.11)	0.148
rs2506350	0.51	0.51 (0.35-1.15)	0.037
rs2506351	0.93	0.93 (0.54-1.64)	0.793
rs2506352	0.82	0.82 (0.47-1.41)	0.491
rs2485534	0.47	0.47 (0.28-0.84)	0.010
rs2485536	0.91	0.91 (0.51-1.55)	0.754
rs2485523	0.82	0.82 (0.47-1.41)	0.491
rs2506362	0.51	0.51 (0.33-0.98)	0.022
rs2506363	0.94	0.94 (0.57-1.72)	0.822
rs10760802	0.50	0.50 (0.34-0.99)	0.018
rs4278209	0.56	0.56 (0.37-1.08)	0.044
**Adjusted by first IVIG used time (days after day one of fever)**			
rs7849782	0.26	0.26 (0.14-0.46)	< 0.001*
rs4742823	0.66	0.66 (0.38-1.11)	0.139
rs2506350	0.64	0.64 (0.35-1.15)	0.141
rs2506351	0.93	0.93 (0.54-1.64)	0.791
rs2506352	0.84	0.84 (0.47-1.41)	0.540
rs2485534	0.49	0.49 (0.28-0.84)	0.011
rs2485536	0.92	0.92 (0.51-1.55)	0.773
rs2485523	0.84	0.84 (0.47-1.41)	0.540
rs2506362	0.57	0.57 (0.33-0.98)	0.045
rs2506363	0.98	0.98 (0.57-1.72)	0.936
rs10760802	0.57	0.57 (0.34-0.99)	0.044
rs4278209	0.63	0.63 (0.37-1.08)	0.095
**Full Model**			
rs7849782	0.25	0.25 (0.14-0.46)	< 0.001*
rs4742823	0.65	0.65 (0.38-1.11)	0.138
rs2506350	0.49	0.49 (0.35-1.15)	0.032
rs2506351	0.93	0.93 (0.54-1.64)	0.818
rs2506352	0.80	0.80 (0.47-1.41)	0.453
rs2485534	0.46	0.46 (0.28-0.84)	0.009
rs2485536	0.89	0.89 (0.51-1.55)	0.703
rs2485523	0.80	0.80 (0.47-1.41)	0.453
rs2506362	0.51	0.51 (0.33-0.98)	0.020
rs2506363	0.94	0.94 (0.57-1.72)	0.831
rs10760802	0.50	0.50 (0.34-0.99)	0.017
rs4278209	0.55	0.55 (0.37-1.08)	0.043

*GRIN3A*, glutamate receptor, ionotropic, N-methyl-D-aspartate 3A; IVIG, Intravenous immunoglobulin; CAA, Coronary artery aneurysm; CI, confidence interval.

Full model shows results from a logistic regression model including the indicated predictors including fever duration (days) and first IVIG used time (days after day one of fever).

## Discussion

In this study, we used a mapping strategy focusing on the *GRIN3A* gene and identified an SNP contributing to the development of CAA formation in Taiwanese children of the Han Chinese background. By using multivariate regression analysis, we observed a significant association between the *GRIN3A* gene polymorphism and the occurrence of CAA in KD patients. The combined frequency of the GG+GC genotypes of *GRIN3A* (rs7849782) was lower in the KD with CAA group than in the KD without CAA group. Our results suggest that the polymorphism of the *GRIN3A* gene may play a role in KD pathogenesis. 

Our results showed that increased CAA formation in KD was associated with clinical risk factors including fever duration and first IVIG used (days after day one of fever). We also categorized the variables “fever duration” and “first IVIG usage” and then examined their relations with CAA by using odds ratios (Tables S8 and S9). A significant difference was observed between KD patients with and without CAA when the fever duration was more than 8 days (Table S8). However, no significant difference was observed between KD patients with CAA and without CAA in terms of first IVIG usage (Table S9). These findings correspond with those shown previously in patients with KD [46–49]. Longer duration of fever and/or a delay in IVIG treatment appear to be risk factors for developing CAA in patients with KD. We also assessed the interaction between the individual SNPs and fever duration and/or the time of first IVIG usage (Tables S10, S11, and S12). We used multiple logistic regression including variables with individual SNPs, fever duration (and/or first IVIG usage), and their interaction. The results suggest that the effects of individual SNPs and the CAA formation of KD were not influenced by fever duration (or first IVIG usage). There seem to be no significant interactions between the gene (*GRIN3A* genetic variations) and environmental (fever duration, delay in IVIG usage) factors.

Our genetic association study showed significant associations between KD with CAA complication, and the *GRIN3A* (rs7849782) genetic variant was observed according to multivariate regression analysis. KD patients with the GG+GC genotypes showed a lower rate of CAA development. These results suggest that the *GRIN3A* gene polymorphism is involved in KD progression. Individuals with KD harboring 1 or 2 copies of the G allele generally did not develop CAA. Therefore, GG or GC genotypes for the *GRIN3A* gene polymorphism may be associated with *GRIN3A* transcript abundance. Results of mammalian genomes analysis suggest that G nucleotides and G-rich sequence elements play an important role in pre-mRNA splicing [19,50,51]. Alteration in the secondary structure of pre-mRNA by nucleotide substitution is another possible mechanism that may influence splicing and mRNA formation [52,53]. Additionally, the SNP identified in this study (rs7849782) showed LD with other SNPs (rs4742823, rs2506350, rs2485534, rs2506362, and rs10760802) (Table S1). *GRIN3A* expression was also shown to be significantly associated with other SNPs (rs4742823, rs2506350, rs2485534, rs2506362, and rs10760802; *p* = 0.0494, 0.03719, 0.03706, 0.02695, and 0.02695, respectively) (http://app3.titan.uio.no/biotools/tool.php?app=snpexp). To investigate the correlation of the SNP rs7849782 genotypes with *GRIN3A* expression, we measured *GRIN3A* mRNA levels by real-time quantitative PCR in peripheral blood mononuclear cells. As shown, the GG+GC genotypes tended to express lower levels of *GRIN3A* than did the other individuals with CC genotype (*p* = 0.020; Figure S1). We therefore used RNA interference to down-regulate *GRIN3A* expression and also used this LPS-induced endothelial cell inflammation model (Figure S2) and firstly showed that *GRIN3A* may regulate endothelial cell inflammation via interference with IL-6 and IL-8 expressions. This is the first study to report that *GRIN3A* is a regulator of vascular inflammation and may be beneficial for many inflammatory diseases associated with endothelial dysfunction.

The glutamate receptor gene, *GRIN3A*, consists of 9 exons and localizes to 9q34. *GRIN3A* codes for the glutamate *N*-methyl-d-aspartate (NMDA) receptor subunit 3A precursor, a 1,115-residue protein representing one of the seven that code for the subunits of *N*-methyl-d-aspartate receptors. These receptors play an essential role at many synapses in the brain, regulating ion flow across membranes in response to glutamate signaling [54]. NMDA receptor expression has been described in barrier forming endothelial cells and the neuroepithelium [29–31]. Furthermore, recent studies have shown that the activation of the NMDA receptor may also activate endothelial cell inflammation and may have important implications in the pathogenesis of immune-mediated vascular diseases [32,33]. KD is a multisystemic disorder with a possible underlying pathology of immune-mediated vasculitis [1,55]. Endothelial cell injury and inflammations are known to be the main mechanisms in the development of KD [4]. When the endothelial cells were stimulated with pathogenic mediators including LPS, the stimulated cells trigger inflammatory signals to increase permeability, leukocyte recruitment [56]. The vascular endothelium is a functional barrier between the vessel wall and bloodstream. In this study, we screened the genetic variants of seven genes that encode subunits of NMDA receptors including *GRIN1*, *GRIN2A*, *GRIN2B*, *GRIN2C*, *GRIN2D*, *GRIN3A* and *GRIN3B*, linked to KD with CAA formation (Table 2, Tables S2, S3, S4, S5, S6 and S7). No significant statistical difference was observed except for the *GRIN3A* (rs7849782) genetic variant by χ^2^ test (*p* < 0.001). Our data suggest that the NMDA receptor molecule-GRIN3A plays a role in CAA development in Taiwanese KD patients. 

In conclusion, our results indicate that *GRIN3A* is significantly associated with KD with CAA complications in Taiwanese children of the Han Chinese ethnic background. Genetic polymorphisms of the *GRIN3A* gene may play a role in KD pathogenesis.

## Supporting Information

Figure S1 **GRIN3A mRNA expression levels in peripheral blood mononuclear cells between the GRIN3A SNP (rs7849782) genotypes.** The relative GRIN3A expression was detected by real-time RT-PCR, and expression from individuals with GG+GC genotypes was compared to that from individuals with CC genotypes. The relative expression levels were expressed as GRIN3A mRNA/ HPRT mRNA ratio.
(TIF)

Figure S2 **Effect of GRIN3A knockdown on IL-6 and IL-8 proinflammatory cytokine mRNA expressions.** HUVEC cells were transfected with siGRIN3A or siNC for 24 h at 37°C followed by 100 µg/mL LPS for another 24 h. A. IL-6 mRNA expression was quantified by RT-qPCR. B. IL-8 mRNA expression was quantified by RT-qPCR. Data represent mean ± SD for three independent experiments.
(TIF)

Table S1 **Analysis of LD among SNPs.**
(DOCX)

Table S2 **Effect of *GRIN1* gene SNPs on the CAA formation in Taiwanese Kawasaki disease patients.**
(DOCX)

Table S3 **Effect of *GRIN2A* gene SNPs on the CAA formation in Taiwanese Kawasaki disease patients.**
(DOCX)

Table S4 **Effect of *GRIN2B* gene SNPs on the CAA formation in Taiwanese Kawasaki disease patients.**
(DOCX)

Table S5 **Effect of *GRIN2C* gene SNPs on the CAA formation in Taiwanese Kawasaki disease patients.**
(DOCX)

Table S6 **Effect of *GRIN2D* gene SNPs on the CAA formation in Taiwanese Kawasaki disease patients.**
(DOCX)

Table S7 **Effect of *GRIN3B* gene SNPs on the CAA formation in Taiwanese Kawasaki disease patients.**
(DOCX)

Table S8 **Distribution of various days of fever duration in KD patients according to the presence or absence of CAA.**
(DOCX)

Table S9 **Distribution of various days of 1st IVIG used time in KD patients according to the presence or absence of CAA.**
(DOCX)

Table S10 **The interaction between fever duration and GRIN3A gene SNPs by using multiple logistic regression analysis.**
(DOCX)

Table S11 **The interaction between 1st IVIG used time and GRIN3A gene SNPs by using multiple logistic regression analysis.**
(DOCX)

Table S12 **The interaction among fever duration, 1st IVIG used time and GRIN3A gene SNPs by using multiple logistic regression analysis.**
(DOCX)
