# Supplementary figures and images for: Association between GRIN3A Gene Polymorphism in Kawasaki Disease and Coronary Artery Aneurysms in Taiwanese Children
Source: PLoS One. 2013 Nov 22;8(11):e81384. doi: 10.1371/journal.pone.0081384 (PMC3838481; doi:10.1371/journal.pone.0081384)

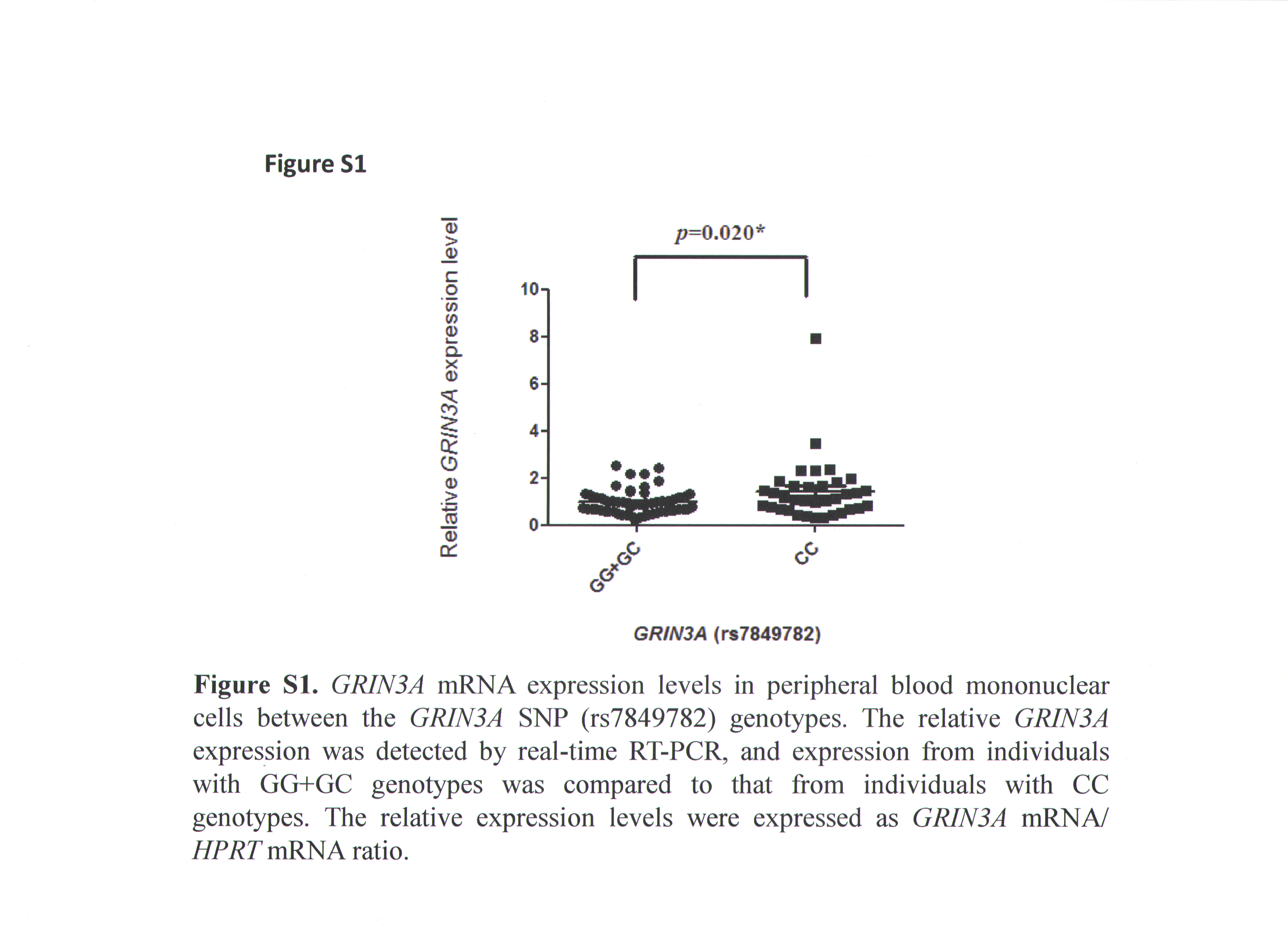

Supplement: Figure S1 — GRIN3A mRNA expression levels in peripheral blood mononuclear cells between the GRIN3A SNP (rs7849782) genotypes. The relative GRIN3A expression was detected by real-time RT-PCR, and expression from individuals with GG+GC genotypes was compared to that from individuals with CC genotypes. The relative expression levels were expressed as GRIN3A mRNA/ HPRT mRNA ratio. (TIF) [file pone.0081384.s001.tif]

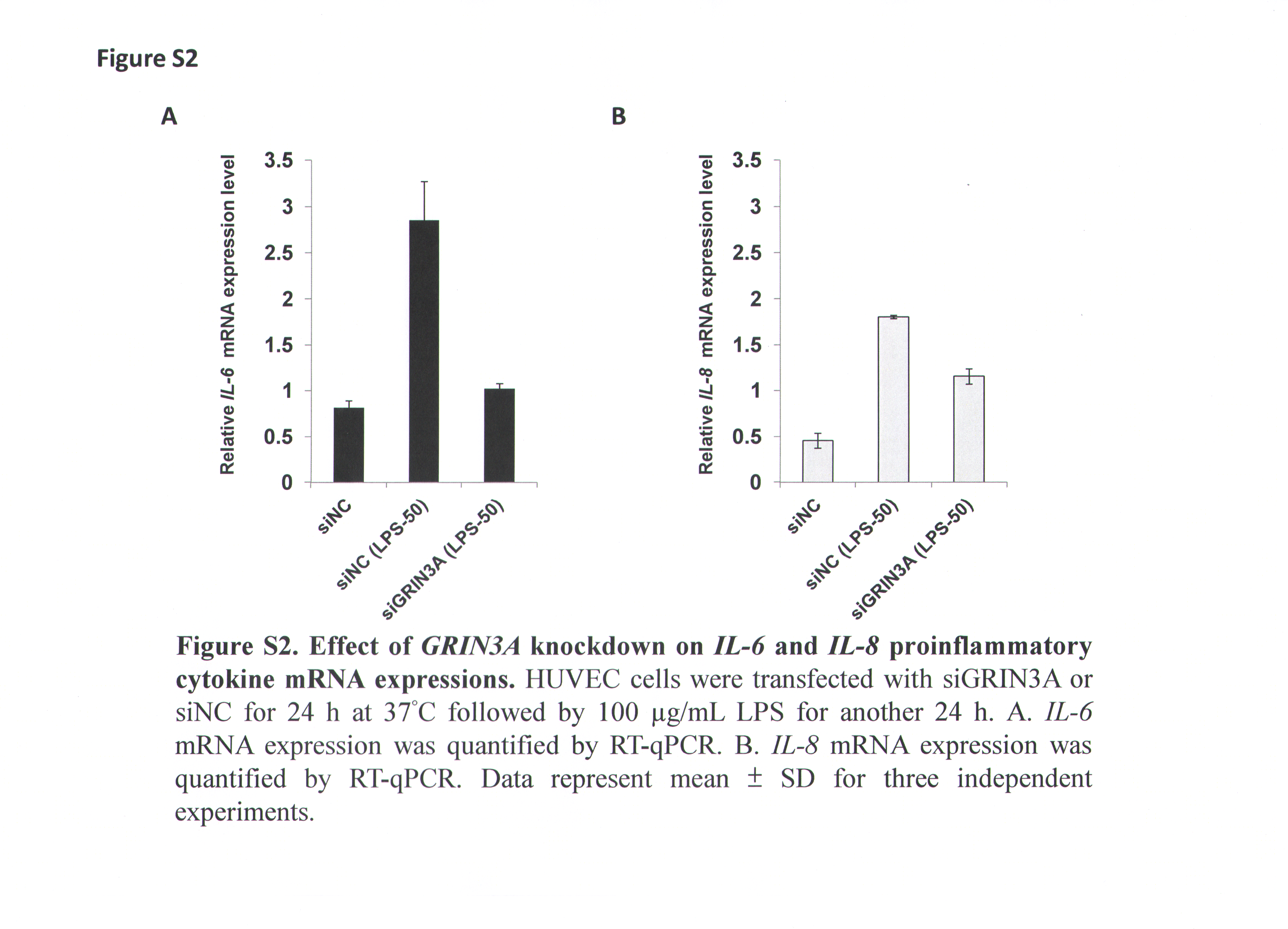

Supplement: Figure S2 — Effect of GRIN3A knockdown on IL-6 and IL-8 proinflammatory cytokine mRNA expressions. HUVEC cells were transfected with siGRIN3A or siNC for 24 h at 37°C followed by 100 µg/mL LPS for another 24 h. A. IL-6 mRNA expression was quantified by RT-qPCR. B. IL-8 mRNA expression was quantified by RT-qPCR. Data represent mean ± SD for three independent experiments. (TIF) [file pone.0081384.s002.tif]
