# Supplementary material for: Association between GRIN3A Gene Polymorphism in Kawasaki Disease and Coronary Artery Aneurysms in Taiwanese Children
Source: PLoS One. 2013 Nov 22;8(11):e81384. doi: 10.1371/journal.pone.0081384 (PMC3838481; doi:10.1371/journal.pone.0081384)
Supplement: Table S1 — Analysis of LD among SNPs. (DOCX) [file pone.0081384.s003.docx]

| **Table S1. Analysis of LD among SNPs** | | | |
| --- | --- | --- | --- |
|  |  |  |  |
|  |  |  |  |
| ***Locus 1*** | ***Locus 2*** | ***D'*** | ***LOD*** |
|  |  |  |  |
|  |  |  |  |
|  |  |  |  |
|  |  |  |  |
| rs7849782 | rs4742823 | 0.83 | 40.71 |
| rs7849782 | rs2506350 | 1.00 | 25.75 |
| rs7849782 | rs2485534 | 1.00 | 61.36 |
| rs7849782 | rs2506362 | 0.76 | 34.86 |
| rs7849782 | rs10760802 | 0.81 | 39.15 |
|  |  |  |  |
|  |  |  |  |
| D' = absolute value of Lewontin's D prime. | | | |
