# Supplementary material for: Association between GRIN3A Gene Polymorphism in Kawasaki Disease and Coronary Artery Aneurysms in Taiwanese Children
Source: PLoS One. 2013 Nov 22;8(11):e81384. doi: 10.1371/journal.pone.0081384 (PMC3838481; doi:10.1371/journal.pone.0081384)
Supplement: Table S2 — Effect of GRIN1 gene SNPs on the CAA formation in Taiwanese Kawasaki disease patients. (DOCX) [file pone.0081384.s004.docx]

| **Table S2. Effect of *GRIN1* gene SNPs on the CAA formation in Taiwanese Kawasaki disease patients** | | | | | | | | | | | | | |
| --- | --- | --- | --- | --- | --- | --- | --- | --- | --- | --- | --- | --- | --- |
| **SNP** | **SNP Chromosome** | **Cytoband** | **Physical Position** | | **Nearest Genes** | |  | **CAA-** | **CAA+** | | | | |
|  |  |  |  | |  | |  | **No. (%)** | **No. (%)** | | ***p* value** | | **Odds ratio (95% CI)** |
| rs12005780 | 9 | q34.3 | 140040905 | | *GRIN1* | | CC+CT | 134 (72.0) | 56 (73.7) | | 0.739 | | 1.09 (0.59-1.99) |
|  |  |  |  | |  | | TT | 52 (28.0) | 20 (26.3) | |  | | 1 |
| rs7019274 | 9 | q34.3 | 140044286 | | *GRIN1* | | AA+AG | 65 (34.9) | 30 (39.5) | | 0.309 | | 1.21 (0.7-2.1) |
|  |  |  |  | |  | | GG | 121 (65.1) | 46 (60.5) | |  | | 1 |
| rs7859352 | 9 | q34.3 | 140055443 | | *GRIN1* | | TT+TC | 108 (58.7) | 39 (53.4) | | 0.543 | | 0.81 (0.47-1.39) |
|  |  |  |  | |  | | CC | 76 (41.3) | 34 (46.6) | |  | | 1 |
| rs7865267 | 9 | q34.3 | 140057896 | | *GRIN1* | | TT+TC | 67 (36.0) | 33 (43.4) | | 0.134 | | 1.36 (0.79-2.35) |
|  |  |  |  | |  | | CC | 119 (64.0) | 43 (56.6) | |  | | 1 |
| rs7021328 | 9 | q34.3 | 140058778 | | *GRIN1* | | TT+TA | 70 (37.6) | 33 (43.4) | | 0.198 | | 1.27 (0.74-2.19) |
|  |  |  |  | |  | | AA | 116 (62.4) | 43 (56.6) | |  | | 1 |
| rs7848769 | 9 | q34.3 | 140059720 | | *GRIN1* | | CC+CG | 67 (36.0) | 31 (40.8) | | 0.251 | | 1.22 (0.71-2.11) |
|  |  |  |  | |  | | GG | 119 (64.0) | 45 (59.2) | |  | | 1 |
|  |  |  |  | |  | |  |  |  | |  | |  |
|  |  |  |  | |  | |  |  |  | |  | |  |
| *GRIN1*, glutamate receptor, ionotropic, N-methyl D-aspartate 1; SNP, single nucleotide polymorphism; CAA, Coronary artery aneurysm; CI, confidence interval. | | | | | | | | | | |  | |  |
| *p*-values were obtained by chi-square test. | | | |  | |  | |  |  |  |  |  |  |
| Bold, emphasizing statistical significance was considered as *p* value <0.0083 (0.05/6). | | | | | | | | | | | | | |
