# Supplementary material for: Association between GRIN3A Gene Polymorphism in Kawasaki Disease and Coronary Artery Aneurysms in Taiwanese Children
Source: PLoS One. 2013 Nov 22;8(11):e81384. doi: 10.1371/journal.pone.0081384 (PMC3838481; doi:10.1371/journal.pone.0081384)
Supplement: Table S3 — Effect of GRIN2A gene SNPs on the CAA formation in Taiwanese Kawasaki disease patients. (DOCX) [file pone.0081384.s005.docx]

| **Table S3. Effect of *GRIN2A* gene SNPs on the CAA formation in Taiwanese Kawasaki disease patients** | | | | | | | | | |
| --- | --- | --- | --- | --- | --- | --- | --- | --- | --- |
| **SNP** | **SNP Chromosome** | **Cytoband** | **Physical Position** | **Nearest Genes** |  | **CAA-** | **CAA+** | | |
|  |  |  |  |  |  | **No. (%)** | **No. (%)** | ***p* value** | **Odds ratio (95% CI)** |
| rs8063193 | 16 | p13.2 | 9855082 | *GRIN2A* | CC+CA | 44 (23.7) | 15 (20.0) | 0.430 | 0.81 (0.42-1.56) |
|  |  |  |  |  | AA | 142 (76.3) | 60 (80.0) |  | 1 |
| rs16966731 | 16 | p13.2 | 9959941 | *GRIN2A* | CC+CT | 59 (31.7) | 31 (40.8) | 0.026 | 1.48 (0.85-2.58) |
|  |  |  |  |  | TT | 127 (68.3) | 45 (59.2) |  | 1 |
| rs837688 | 16 | p13.2 | 10007044 | *GRIN2A* | CC+CA | 69 (37.7) | 31 (41.9) | 0.163 | 1.19 (0.69-2.06) |
|  |  |  |  |  | AA | 114 (62.3) | 43 (58.1) |  | 1 |
| rs10500373 | 16 | p13.2 | 10032977 | *GRIN2A* | CC+CT | 113 (60.8) | 50 (65.8) | 0.749 | 1.24 (0.71-2.17) |
|  |  |  |  |  | TT | 73 (39.2) | 26 (34.2) |  | 1 |
| rs7192395 | 16 | p13.2 | 10110303 | *GRIN2A* | GG+GA | 128 (70.7) | 55 (73.3) | 0.990 | 1.14 (0.62-2.08) |
|  |  |  |  |  | AA | 53 (29.3) | 20 (26.7) |  | 1 |
| rs6497731 | 16 | p13.2 | 10153889 | *GRIN2A* | GG+GC | 136 (73.1) | 57 (76.0) | 0.744 | 1.16 (0.63-2.17) |
|  |  |  |  |  | CC | 50 (26.9) | 18 (24.0) |  | 1 |
| rs1102309 | 16 | p13.2 | 10189632 | *GRIN2A* | TT+TC | 22 (11.8) | 6 (7.9) | 0.497 | 0.64 (0.25-1.64) |
|  |  |  |  |  | CC | 164 (88.2) | 70 (92.1) |  | 1 |
| rs1074368 | 16 | p13.2 | 10221504 | *GRIN2A* | CC+CT | 79 (42.7) | 28 (36.8) | 0.614 | 0.78 (0.45-1.36) |
|  |  |  |  |  | TT | 106 (57.3) | 48 (63.2) |  | 1 |
| rs10852286 | 16 | p13.2 | 10264641 | *GRIN2A* | GG+GA | 115 (62.2) | 38 (50.0) | 0.184 | 0.61 (0.36-1.04) |
|  |  |  |  |  | AA | 70 (37.8) | 38 (50.0) |  | 1 |
|  |  |  |  |  |  |  |  |  |  |
|  |  |  |  |  |  |  |  |  |  |
| *GRIN2A*, glutamate receptor, ionotropic, N-methyl D-aspartate 2A; SNP, single nucleotide polymorphism; CAA, Coronary artery aneurysm; CI, confidence interval. | | | | | | | |  |  |
| *p*-values were obtained by chi-square test. | | |  |  |  |  |  |  |  |
| Bold, emphasizing statistical significance was considered as *p* value <0.0056 (0.05/9). | | | | | | | | | |
