# Supplementary material for: Association between GRIN3A Gene Polymorphism in Kawasaki Disease and Coronary Artery Aneurysms in Taiwanese Children
Source: PLoS One. 2013 Nov 22;8(11):e81384. doi: 10.1371/journal.pone.0081384 (PMC3838481; doi:10.1371/journal.pone.0081384)
Supplement: Table S4 — Effect of GRIN2B gene SNPs on the CAA formation in Taiwanese Kawasaki disease patients. (DOCX) [file pone.0081384.s006.docx]

| **Table S4. Effect of *GRIN2B* gene SNPs on the CAA formation in Taiwanese Kawasaki disease patients** | | | | | | | | | |
| --- | --- | --- | --- | --- | --- | --- | --- | --- | --- |
| **SNP** | **SNP Chromosome** | **Cytoband** | **Physical Position** | **Nearest Genes** |  | **CAA-** | **CAA+** | | |
|  |  |  |  |  |  | **No. (%)** | **No. (%)** | ***p* value** | **Odds ratio (95% CI)** |
| rs2300245 | 12 | p12 | 13732562 | *GRIN2B* | GG+GT | 116 (62.4) | 46 (60.5) | 0.447 | 0.93 (0.54-1.6) |
|  |  |  |  |  | TT | 70 (37.6) | 30 (39.5) |  | 1 |
| rs2284410 | 12 | p12 | 13751078 | *GRIN2B* | TT+TA | 50 (26.9) | 24 (31.6) | 0.802 | 1.26 (0.7-2.25) |
|  |  |  |  |  | AA | 136 (73.1) | 52 (68.4) |  | 1 |
| rs2268122 | 12 | p12 | 13788728 | *GRIN2B* | TT+TG | 87 (47.3) | 39 (51.3) | 0.470 | 1.18 (0.69-2.01) |
|  |  |  |  |  | GG | 97 (52.7) | 37 (48.7) |  | 1 |
| rs11055621 | 12 | p12 | 13835826 | *GRIN2B* | CC+CT | 60 (32.4) | 28 (36.8) | 0.485 | 1.22 (0.7-2.12) |
|  |  |  |  |  | TT | 125 (67.6) | 48 (63.2) |  | 1 |
| rs1861788 | 12 | p12 | 13891859 | *GRIN2B* | GG+GA | 75 (41.0) | 28 (37.3) | 0.529 | 0.86 (0.49-1.49) |
|  |  |  |  |  | AA | 108 (59.0) | 47 (62.7) |  | 1 |
| rs10492141 | 12 | p12 | 13936517 | *GRIN2B* | GG+GA | 76 (41.1) | 25 (32.9) | 0.205 | 0.70 (0.4-1.23) |
|  |  |  |  |  | AA | 109 (58.9) | 51 (67.1) |  | 1 |
| rs10845862 | 12 | p12 | 13968903 | *GRIN2B* | AA+AG | 116 (62.4) | 50 (65.8) | 0.483 | 1.16 (0.66-2.03) |
|  |  |  |  |  | GG | 70 (37.6) | 26 (34.2) |  | 1 |
| rs219920 | 12 | p12 | 13992578 | *GRIN2B* | TT+TC | 132 (71.4) | 56 (73.7) | 0.627 | 1.12 (0.62-2.05) |
|  |  |  |  |  | CC | 53 (28.6) | 20 (26.3) |  | 1 |
| rs11055719 | 12 | p12 | 14055747 | *GRIN2B* | AA+AC | 86 (46.2) | 39 (51.3) | 0.587 | 1.23 (0.72-2.09) |
|  |  |  |  |  | CC | 100 (53.8) | 37 (48.7) |  | 1 |
| *GRIN2B*, glutamate receptor, ionotropic, N-methyl D-aspartate 2B; SNP, single nucleotide polymorphism; CAA, Coronary artery aneurysm; CI, confidence interval. | | | | | | | |  |  |
| *p*-values were obtained by chi-square test. | | |  |  |  |  |  |  |  |
| Bold, emphasizing statistical significance was considered as *p* value <0.0056 (0.05/9). | | | | | | | | | |
