# Supplementary material for: Association between GRIN3A Gene Polymorphism in Kawasaki Disease and Coronary Artery Aneurysms in Taiwanese Children
Source: PLoS One. 2013 Nov 22;8(11):e81384. doi: 10.1371/journal.pone.0081384 (PMC3838481; doi:10.1371/journal.pone.0081384)
Supplement: Table S5 — Effect of GRIN2C gene SNPs on the CAA formation in Taiwanese Kawasaki disease patients. (DOCX) [file pone.0081384.s007.docx]

| **Table S5. Effect of *GRIN2C* gene SNPs on the CAA formation in Taiwanese Kawasaki disease patients** | | | | | | | | | |
| --- | --- | --- | --- | --- | --- | --- | --- | --- | --- |
| **SNP** | **SNP Chromosome** | **Cytoband** | **Physical Position** | **Nearest Genes** |  | **CAA-** | **CAA+** | | |
|  |  |  |  |  |  | **No. (%)** | **No. (%)** | ***p* value** | **Odds ratio (95% CI)** |
| rs2411109 | 17 | q25.1 | 72844051 | *GRIN2C* | GG+GT | 133 (71.5) | 57 (75.0) | 0.624 | 1.20 (0.65-2.2) |
|  |  |  |  |  | TT | 53 (28.5) | 19 (25.0) |  | 1 |
| rs873370 | 17 | q25.1 | 72852999 | *GRIN2C* | TT+TA | 70 (37.6) | 19 (25.0) | 0.095 | 0.55 (0.3-1) |
|  |  |  |  |  | AA | 116 (62.4) | 57 (75.0) |  | 1 |
|  |  |  |  |  |  |  |  |  |  |
|  |  |  |  |  |  |  |  |  |  |
| *GRIN2C*, glutamate receptor, ionotropic, N-methyl D-aspartate 2C; SNP, single nucleotide polymorphism; CAA, Coronary artery aneurysm; CI, confidence interval. | | | | | | | |  |  |
| *p*-values were obtained by chi-square test. | | |  |  |  |  |  |  |  |
| Bold, emphasizing statistical significance was considered as *p* value <0.025 (0.05/2). | | | | | | | | | |
