# Supplementary material for: Association between GRIN3A Gene Polymorphism in Kawasaki Disease and Coronary Artery Aneurysms in Taiwanese Children
Source: PLoS One. 2013 Nov 22;8(11):e81384. doi: 10.1371/journal.pone.0081384 (PMC3838481; doi:10.1371/journal.pone.0081384)
Supplement: Table S6 — Effect of GRIN2D gene SNPs on the CAA formation in Taiwanese Kawasaki disease patients. (DOCX) [file pone.0081384.s008.docx]

| **Table S6. Effect of *GRIN2D* gene SNPs on the CAA formation in Taiwanese Kawasaki disease patients** | | | | | | | | | |
| --- | --- | --- | --- | --- | --- | --- | --- | --- | --- |
| **SNP** | **SNP Chromosome** | **Cytoband** | **Physical Position** | **Nearest Genes** |  | **CAA-** | **CAA+** | | |
|  |  |  |  |  |  | **No. (%)** | **No. (%)** | ***p* value** | **Odds ratio (95% CI)** |
| rs538215 | 19 | q13.33 | 48901641 | *GRIN2D* | TT+TC | 134 (73.6) | 56 (75.7) | 0.270 | 1.11 (0.60-2.08) |
|  |  |  |  |  | CC | 48 (26.4) | 18 (24.3) |  | 1 |
| rs380442 | 19 | q13.33 | 48905894 | *GRIN2D* | TT+TG | 66 (35.7) | 36 (47.4) | 0.077 | 1.62 (0.94-2.79) |
|  |  |  |  |  | GG | 119 (64.3) | 40 (52.6) |  | 1 |
| rs10410645 | 19 | q13.33 | 48906580 | *GRIN2D* | AA+AC | 107 (58.5) | 47 (62.7) | 0.184 | 1.19 (0.69-2.07) |
|  |  |  |  |  | CC | 76 (41.5) | 28 (37.3) |  | 1 |
| rs2074634 | 19 | q13.33 | 48930187 | *GRIN2D* | CC+CT | 64 (34.8) | 24 (31.6) | 0.843 | 0.87 (0.49-1.53) |
|  |  |  |  |  | TT | 120 (65.2) | 52 (68.4) |  | 1 |
| rs173202 | 19 | q13.33 | 48945428 | *GRIN2D* | TT+TC | 88 (47.3) | 37 (48.7) | 0.670 | 1.06 (0.62-1.8) |
|  |  |  |  |  | CC | 98 (52.7) | 39 (51.3) |  | 1 |
| rs346544 | 19 | q13.33 | 48948045 | *GRIN2D* | GG+GA | 88 (47.3) | 37 (48.7) | 0.670 | 1.06 (0.62-1.8) |
|  |  |  |  |  | AA | 98 (52.7) | 39 (51.3) |  | 1 |
|  |  |  |  |  |  |  |  |  |  |
|  |  |  |  |  |  |  |  |  |  |
| *GRIN2D*,glutamate receptor, ionotropic, N-methyl D-aspartate 2D; SNP, single nucleotide polymorphism; CAA, Coronary artery aneurysm; CI, confidence interval. | | | | | | | |  |  |
| *p*-values were obtained by chi-square test. | | |  |  |  |  |  |  |  |
| Bold, emphasizing statistical significance was considered as *p* value <0.0083 (0.05/6). | | | | | | | | | |
