# Supplementary material for: Association between GRIN3A Gene Polymorphism in Kawasaki Disease and Coronary Artery Aneurysms in Taiwanese Children
Source: PLoS One. 2013 Nov 22;8(11):e81384. doi: 10.1371/journal.pone.0081384 (PMC3838481; doi:10.1371/journal.pone.0081384)
Supplement: Table S7 — Effect of GRIN3B gene SNPs on the CAA formation in Taiwanese Kawasaki disease patients. (DOCX) [file pone.0081384.s009.docx]

| **Table S7. Effect of *GRIN3B* gene SNPs on the CAA formation in Taiwanese Kawasaki disease patients** | | | | | | | | | |
| --- | --- | --- | --- | --- | --- | --- | --- | --- | --- |
| **SNP** | **SNP Chromosome** | **Cytoband** | **Physical Position** | **Nearest Genes** |  | **CAA-** | **CAA+** | | |
|  |  |  |  |  |  | **No. (%)** | **No. (%)** | ***p* value** | **Odds ratio (95% CI)** |
| rs3752243 | 19 | p13.3 | 1005060 | *GRIN3B* | CC+CT | 116 (63.4) | 43 (58.1) | 0.341 | 0.80 (0.46-1.39) |
|  |  |  |  |  | TT | 67 (36.6) | 31 (41.9) |  | 1 |
| rs4147918 | 19 | p13.3 | 1009176 | *GRIN3B* | GG+GA | 50 (26.9) | 27 (35.5) | 0.281 | 1.50 (0.85-2.65) |
|  |  |  |  |  | AA | 136 (73.1) | 49 (64.5) |  | 1 |
|  |  |  |  |  |  |  |  |  |  |
|  |  |  |  |  |  |  |  |  |  |
| *GRIN3B*, glutamate receptor, ionotropic, N-methyl-D-aspartate 3B; SNP, single nucleotide polymorphism; CAA, Coronary artery aneurysm; CI, confidence interval. | | | | | | | |  |  |
| *p*-values were obtained by chi-square test. | | |  |  |  |  |  |  |  |
| Bold, emphasizing statistical significance was considered as *p* value <0.025 (0.05/2). | | | | | | | | | |
