# Supplementary material for: Association between GRIN3A Gene Polymorphism in Kawasaki Disease and Coronary Artery Aneurysms in Taiwanese Children
Source: PLoS One. 2013 Nov 22;8(11):e81384. doi: 10.1371/journal.pone.0081384 (PMC3838481; doi:10.1371/journal.pone.0081384)
Supplement: Table S8 — Distribution of various days of fever duration in KD patients according to the presence or absence of CAA. (DOCX) [file pone.0081384.s010.docx]

| **Table S8. Distribution of various days of fever duration in KD patients according to the presence or absence of CAA** | | | | | |
| --- | --- | --- | --- | --- | --- |
| **Fever duration (days)** | **Kawasaki disease** | | **Odds ratio** | **95% CI** | ***p* value** |
|  | **CAA- (Numbers (%))** | **CAA+ (Numbers (%))** |  |  |  |
| Cut points for 3 equal groups^a^ |  |  |  |  |  |
| 5 days | 33 (75.0%) | 11 (25.0%) | 1 | - |  |
| 6 ≦ Fever duration < 8 | 91 (82.7%) | 19 (17.3%) | 0.63 | (0.270-1.455) | 0.2765 |
| ≧ 8 days | 62 (57.4%) | 46 (42.6%) | 2.22 | (1.019-4.864) | 0.0448 |
| Combined into 2 groups^b^ |  |  |  |  |  |
| 5 ≦ Fever duration < 8 | 124 (80.5%) | 30 (19.5%) | 1 |  |  |
| ≧ 8 days | 62 (57.4%) | 46 (42.6%) | 3.07 | (1.766-5.325) | ***< 0.0001*** |
| 95% CI, 95% confidence interval; KD, kawasaki disease; CAA, coronary artery aneurysm. | | |  |  |  |
| ^a^The KD patients were divided into 3 equal groups (the 33.33th percentile is 6 days (fever duration) and the 66.66th percentile is 8 days of fever duration). The *p* value, odds ratio and 95% CI were obtained by 2 x 3 chi-square test. | | | | | |
| ^b^ The KD patients were also combined into 2 groups ( the KD patients with 5 ≦ Fever duration < 8; the KD patients with fever duration more than 8 days). The *p* value, odds ratio and 95% CI were obtained by 2 x 2 chi-square test. | | | | | |
