# Supplementary material for: Association between GRIN3A Gene Polymorphism in Kawasaki Disease and Coronary Artery Aneurysms in Taiwanese Children
Source: PLoS One. 2013 Nov 22;8(11):e81384. doi: 10.1371/journal.pone.0081384 (PMC3838481; doi:10.1371/journal.pone.0081384)
Supplement: Table S9 — Distribution of various days of 1st IVIG used time in KD patients according to the presence or absence of CAA. (DOCX) [file pone.0081384.s011.docx]

| **Table S9. Distribution of various days of 1st IVIG used time in KD patients according to the presence or absence of CAA** | | | | | |
| --- | --- | --- | --- | --- | --- |
| **1st IVIG used time (days after the first date with fever)** | **Kawasaki disease** | | **Odds ratio** | **95% CI** | ***p* value** |
|  | **CAA- (Numbers (%))** | **CAA+ (Numbers (%))** |  |  |  |
| Cut points for 3 equal groups^a^ |  |  |  |  |  |
| 1 < 1st IVIG used time ≦ 4 | 21 (72.4%) | 8 (27.6%) | 1 | - |  |
| 5 days | 55 (75.3%) | 18 (24.7%) | 0.86 | (0.325-2.273) | 0.760 |
| ≧ 6 days | 110 (68.8%) | 50 (31.2%) | 1.19 | (0.495-2.877) | 0.694 |
| Combined into 2 groups^b^ |  |  |  |  |  |
| 1 < 1st IVIG used time ≦ 5 | 76 (74.5%) | 26 (25.5%) | 1 | - |  |
| ≧ 6 days | 110 (68.8%) | 50 (31.2%) | 1.33 | (0.761-2.319) | 0.317 |
| IVIG, intravenous immunoglobulin; 95% CI, 95% confidence interval; KD, kawasaki disease; CAA, coronary artery aneurysm. | | | | |  |
| ^a^The KD patients were divided into 3 equal groups (the 33.33th percentile is 5 days (1st IVIG used time) and the 66.66th percentile is 6 days of 1st IVIG used time). The *p* value, odds ratio and 95% CI were obtained by 2 x 3 chi-square test. | | | | | |
| ^b^ The KD patients were also combined into 2 groups ( the KD patients with 1 < 1st IVIG used time ≦ 5; the KD patients with 1st IVIG used time more than 6 days). The *p* value, odds ratio and 95% CI were obtained by 2 x 2 chi-square test. | | | | | |
