# Supplementary material for: Association between GRIN3A Gene Polymorphism in Kawasaki Disease and Coronary Artery Aneurysms in Taiwanese Children
Source: PLoS One. 2013 Nov 22;8(11):e81384. doi: 10.1371/journal.pone.0081384 (PMC3838481; doi:10.1371/journal.pone.0081384)
Supplement: Table S10 — The interaction between fever duration and GRIN3A gene SNPs by using multiple logistic regression analysis. (DOCX) [file pone.0081384.s012.docx]

| **Table S10. The interaction between fever duration and *GRIN3A* gene SNPs by using multiple logistic regression analysis** | | | | | | | | |
| --- | --- | --- | --- | --- | --- | --- | --- | --- |
| **CHR** | **SNP** | **POSITION** | **A1** | **TEST** | **NMISS** | **OR** | **STAT** | ***P*** |
| 9 | rs7849782 | 103467085 | G | DOMxCOV1 | 262 | 2.37 | 1.416 | 0.157 |
| 9 | rs4742823 | 103481593 | C | DOMxCOV1 | 262 | 1.759 | 0.9884 | 0.323 |
| 9 | rs2506350 | 103482467 | T | DOMxCOV1 | 262 | 2.628 | 1.384 | 0.166 |
| 9 | rs2506351 | 103482557 | C | DOMxCOV1 | 262 | 0.6994 | -0.606 | 0.545 |
| 9 | rs2506352 | 103483140 | A | DOMxCOV1 | 262 | 1.441 | 0.6338 | 0.526 |
| 9 | rs2485534 | 103491159 | T | DOMxCOV1 | 257 | 1.549 | 0.7463 | 0.456 |
| 9 | rs2485536 | 103491461 | A | DOMxCOV1 | 261 | 1.163 | 0.2582 | 0.796 |
| 9 | rs2485523 | 103497057 | G | DOMxCOV1 | 262 | 1.441 | 0.6338 | 0.526 |
| 9 | rs2506362 | 103516083 | A | DOMxCOV1 | 261 | 1.165 | 0.2657 | 0.791 |
| 9 | rs2506363 | 103516551 | C | DOMxCOV1 | 262 | 0.8287 | -0.3207 | 0.748 |
| 9 | rs10760802 | 103520656 | T | DOMxCOV1 | 262 | 1.306 | 0.4654 | 0.642 |
| 9 | rs4278209 | 103535011 | A | DOMxCOV1 | 261 | 1.021 | 0.03576 | 0.972 |
