# Supplementary material for: Association between GRIN3A Gene Polymorphism in Kawasaki Disease and Coronary Artery Aneurysms in Taiwanese Children
Source: PLoS One. 2013 Nov 22;8(11):e81384. doi: 10.1371/journal.pone.0081384 (PMC3838481; doi:10.1371/journal.pone.0081384)
Supplement: Table S11 — The interaction between 1st IVIG used time and GRIN3A gene SNPs by using multiple logistic regression analysis. (DOCX) [file pone.0081384.s013.docx]

| **Table S11. The interaction between 1st IVIG used time and *GRIN3A* gene SNPs by using multiple logistic regression analysis** | | | | | | | | |
| --- | --- | --- | --- | --- | --- | --- | --- | --- |
| **CHR** | **SNP** | **POSITION** | **A1** | **TEST** | **NMISS** | **OR** | **STAT** | ***P*** |
| 9 | rs7849782 | 103467085 | G | DOMxCOV1 | 262 | 1.236 | 0.346 | 0.729 |
| 9 | rs4742823 | 103481593 | C | DOMxCOV1 | 262 | 1.311 | 0.4715 | 0.637 |
| 9 | rs2506350 | 103482467 | T | DOMxCOV1 | 262 | 1.134 | 0.1962 | 0.845 |
| 9 | rs2506351 | 103482557 | C | DOMxCOV1 | 262 | 0.5851 | -0.9043 | 0.366 |
| 9 | rs2506352 | 103483140 | A | DOMxCOV1 | 262 | 1.087 | 0.1433 | 0.886 |
| 9 | rs2485534 | 103491159 | T | DOMxCOV1 | 257 | 1.338 | 0.4995 | 0.617 |
| 9 | rs2485536 | 103491461 | A | DOMxCOV1 | 261 | 0.9672 | -0.05611 | 0.955 |
| 9 | rs2485523 | 103497057 | G | DOMxCOV1 | 262 | 1.087 | 0.1433 | 0.886 |
| 9 | rs2506362 | 103516083 | A | DOMxCOV1 | 261 | 1.565 | 0.7746 | 0.439 |
| 9 | rs2506363 | 103516551 | C | DOMxCOV1 | 262 | 0.3094 | -1.921 | 0.055 |
| 9 | rs10760802 | 103520656 | T | DOMxCOV1 | 262 | 1.568 | 0.7785 | 0.436 |
| 9 | rs4278209 | 103535011 | A | DOMxCOV1 | 261 | 1.981 | 1.178 | 0.239 |
