# Supplementary material for: Association between GRIN3A Gene Polymorphism in Kawasaki Disease and Coronary Artery Aneurysms in Taiwanese Children
Source: PLoS One. 2013 Nov 22;8(11):e81384. doi: 10.1371/journal.pone.0081384 (PMC3838481; doi:10.1371/journal.pone.0081384)
Supplement: Table S12 — The interaction among fever duration, 1st IVIG used time and GRIN3A gene SNPs by using multiple logistic regression analysis. (DOCX) [file pone.0081384.s014.docx]

| **Table S12. The interaction among fever duration, 1st IVIG used time and *GRIN3A* gene SNPs by using multiple logistic regression analysis** | | | | | | | | |
| --- | --- | --- | --- | --- | --- | --- | --- | --- |
| **CHR** | **SNP** | **POSITION** | **A1** | **TEST** | **NMISS** | **OR** | **STAT** | ***P*** |
| 9 | rs7849782 | 103467085 | G | DOMxCOV1 | 262 | 2.59 | 1.506 | 0.132 |
| 9 | rs7849782 | 103467085 | G | DOMxCOV2 | 262 | 0.756 | -0.431 | 0.666 |
| 9 | rs4742823 | 103481593 | C | DOMxCOV1 | 262 | 1.728 | 0.919 | 0.358 |
| 9 | rs4742823 | 103481593 | C | DOMxCOV2 | 262 | 1.078 | 0.123 | 0.903 |
| 9 | rs2506350 | 103482467 | T | DOMxCOV1 | 262 | 2.526 | 1.3 | 0.194 |
| 9 | rs2506350 | 103482467 | T | DOMxCOV2 | 262 | 1.23 | 0.298 | 0.766 |
| 9 | rs2506351 | 103482557 | C | DOMxCOV1 | 262 | 0.81 | -0.345 | 0.731 |
| 9 | rs2506351 | 103482557 | C | DOMxCOV2 | 262 | 0.56 | -0.916 | 0.360 |
| 9 | rs2506352 | 103483140 | A | DOMxCOV1 | 262 | 1.376 | 0.523 | 0.601 |
| 9 | rs2506352 | 103483140 | A | DOMxCOV2 | 262 | 1.149 | 0.218 | 0.827 |
| 9 | rs2485534 | 103491159 | T | DOMxCOV1 | 257 | 1.471 | 0.632 | 0.528 |
| 9 | rs2485534 | 103491159 | T | DOMxCOV2 | 257 | 1.207 | 0.3 | 0.765 |
| 9 | rs2485536 | 103491461 | A | DOMxCOV1 | 261 | 1.123 | 0.187 | 0.852 |
| 9 | rs2485536 | 103491461 | A | DOMxCOV2 | 261 | 1.09 | 0.133 | 0.895 |
| 9 | rs2485523 | 103497057 | G | DOMxCOV1 | 262 | 1.376 | 0.523 | 0.601 |
| 9 | rs2485523 | 103497057 | G | DOMxCOV2 | 262 | 1.149 | 0.218 | 0.827 |
| 9 | rs2506362 | 103516083 | A | DOMxCOV1 | 261 | 1.022 | 0.036 | 0.971 |
| 9 | rs2506362 | 103516083 | A | DOMxCOV2 | 261 | 1.581 | 0.731 | 0.465 |
| 9 | rs2506363 | 103516551 | C | DOMxCOV1 | 262 | 1.148 | 0.226 | 0.821 |
| 9 | rs2506363 | 103516551 | C | DOMxCOV2 | 262 | 0.265 | -2.045 | 0.041 |
| 9 | rs10760802 | 103520656 | T | DOMxCOV1 | 262 | 1.159 | 0.246 | 0.805 |
| 9 | rs10760802 | 103520656 | T | DOMxCOV2 | 262 | 1.547 | 0.703 | 0.482 |
| 9 | rs4278209 | 103535011 | A | DOMxCOV1 | 261 | 0.812 | -0.345 | 0.730 |
| 9 | rs4278209 | 103535011 | A | DOMxCOV2 | 261 | 2.24 | 1.282 | 0.200 |
